# The Slow Pandemic: Emergence of Antimicrobial Resistance in the Postadvent of SARS-CoV-2 Pandemic

**DOI:** 10.1155/ghe3/3172234

**Published:** 2025-04-16

**Authors:** Ayodeji Osunla, Femi Oloye, Adeoye Kayode, Oluwabunmi Femi-Oloye, Ayomide Okiti, Mark Servos, John Giesy

**Affiliations:** ^1^Toxicology Centre, University of Saskatchewan, Saskatoon, Saskatchewan, Canada; ^2^Department of Microbiology, Adekunle Ajasin University, Akungba-Akoko, Ondo, Nigeria; ^3^Division of Physical and Computational Sciences, University of Pittsburgh, Bradford, Pennsylvania, USA; ^4^Department of Biochemistry, Genetics, and Microbiology, Forestry and Agricultural Biotechnology Institute, University of Pretoria, Pretoria, South Africa; ^5^Department of Biology, University of Waterloo, Waterloo, Ontario, Canada; ^6^Department of Veterinary Biomedical Sciences, University of Saskatchewan, Saskatoon, Saskatchewan, Canada; ^7^Department of Integrative Biology and Centre for Integrative Toxicology, Michigan State University, East Lansing, Michigan, USA

**Keywords:** antimicrobial resistance, antiseptic and pathogen, disinfectants, quaternary ammonium compounds, SARS-CoV-2 pandemic

## Abstract

**Background:** The unprecedented outbreak of the severe acute respiratory syndrome coronavirus 2 (SARS-CoV-2) pandemic has dramatically changed the global approach to public health, emphasizing the importance of measures to control and prevent infections. In response to the COVID-19 crisis, stringent hygiene practices and surface disinfection have become the norm, with an unprecedented surge in the use of disinfectants and antiseptics (DAs).

**Main Text:** While these measures have been crucial in curbing the spread of the virus, an emerging concern has taken center stage: the potential impact of the prolonged and widespread use of antimicrobial compounds in these products on the development of antibiotic resistance. Antimicrobial resistance (AMR) has long been recognized as one of the most pressing global health threats. Quaternary ammonium compounds (QAC) such as benzalkonium chloride, benzethonium chloride, and cetylpyridinium chloride, which are extensively used in DAs formulations, have gained less attention in the context of AMR.

**Conclusion:** A high abundance of QACs was detected in wastewater, and certain bacteria such as *Pseudomonas aeruginosa, Acinetobacter baumannii*, and *Enterococcus* species developed resistance to these compounds over time. We analyzed the available evidence from the scientific literature, examining the presence and concentrations of QACs in different water sources, and their resistance mechanisms. This review aimed to shed light on the multifaceted challenges that arise from the dual battle against the COVID-19 pandemic and the ongoing global fight against AMR.

## 1. Introduction

In an era characterized by remarkable medical advancements and improved life expectancy, the shadow of antimicrobial resistance (AMR) looms over the progress made by the introduction of antibiotics and synthetic antimicrobials. AMR is an escalating global challenge that jeopardizes the progress made in modern healthcare, making routine infections difficult to treat and turning once-treatable illnesses into potentially fatal threats [1]. This phenomenon occurs when microorganisms, including bacteria, viruses, fungi, and parasites, acquire resistance to antibiotics intended to eradicate them, thereby diminishing the efficacy of therapeutic interventions [2]. The emergence of AMR can be attributed to the excessive and inappropriate use of antimicrobial agents, including antibiotics, across the human, animal, and agricultural domains [3]. Extended exposure to these agents provides opportunities for microbes to adapt and evolve, thereby leading to the development of mechanisms that counteract the effects of medications. Consequently, the difficulty in managing infections escalates, resulting in extended periods of illness, elevated healthcare expenditure, and an increased likelihood of mortality [4]. The World Health Organization (WHO) has recognized AMR as a significant peril to global public health, necessitating immediate attention and collaborative efforts from states worldwide [5].

Incorrect utilization of antibiotics is a significant contributing factor to the dissemination of AMR. Patients commonly exhibit nonadherence to recommended antibiotic regimens and prescribing antibiotics for viral infections despite their lack of efficacy in such cases. The use of antibiotics in agricultural practices to enhance the growth of animals exacerbates this issue [6]. These practices facilitate the development and spread of drug resistance in microorganisms. The consequences of AMR are far-reaching, affecting not only individual health, but also global economies and healthcare systems [7]. Frequently performed medical operations, such as surgery, chemotherapy, and organ transplants, might pose significant risks among immune-deficient individuals because of their heightened vulnerability to infections [4]. The financial implications of managing resistant infections in healthcare are significantly elevated, placing extra pressure on already overwhelmed health systems and perhaps exacerbating the economic vulnerability of marginalized groups.

The outbreak of the COVID-19 pandemic elicited an unprecedented global response, characterized by a significant emphasis on preventing infection and implementing control measures to curb the spread of the virus [8]. One of the most widely adopted strategies is to increase the use of DAs in public spaces, healthcare facilities, and homes. These measures are crucial in reducing the transmission of the virus; however, there is growing concern that excessive and inappropriate use of these agents could inadvertently contribute to the evolution of AMR [9]. DAs are chemical substances specifically formulated to eradicate or impede the proliferation of bacteria on various surfaces and on the skin. They play a vital role in reducing the risk of infections in healthcare settings and their significance has become even more apparent during pandemics [9]. However, the overuse or misuse of these agents can result in unintended consequences. When DAs are used, or when pathogens are exposed to sublethal concentrations of these chemicals, not all microorganisms can be eliminated or completely killed, which allows them to develop new ways to survive. This could be achieved by increasing the activity of efflux pumps, biofilm formation, or other defense mechanisms that make bacteria less susceptible to both antibiotics and antiseptics. Some microorganisms may survive because of their natural or acquired resistance to genetic mutations. Furthermore, repeated exposure to these chemicals can lead to the selection for and proliferation of resistant strains of microorganisms, which is a significant concern in healthcare settings and beyond [10]. This phenomenon underscores the importance of judicious use of such agents to help combat the growing problem of AMR. For instance, benzethonium chloride, benzalkonium chloride, and cetylpyridinium chloride are quaternary ammonium compounds (QAC) often found in DAs mixtures [9]. In addition to antibiotics and biocides, bacteria can become resistant to other harmful environmental compounds found in the soil, water, food, and feed [11]. The public is continually bombarded with messages in various media, encouraging them to purchase and use antimicrobials during all phases of their lives. Although these chemicals have been shown to kill or slow the growth of bacteria, their unnecessary or long-term use might facilitate the development of AMR in the future. Therefore, this review intends to uncover the multifaceted challenges that arise from the dual battle against the COVID-19 pandemic and the ongoing global fight against AMR, and to elucidate the mechanisms underlying the development of co-resistance.

## 2. Role of QAC in the Efficacy of Antiseptics and Disinfectants

DAs play crucial roles in maintaining hygiene standards, mitigating the spread of infections, and controlling microbial growth in various settings such as healthcare facilities, households, food industries, and paper industries [12, 13]. QACs are a broad class of positively charged nitrogen-containing chemicals employed as hydrophobic integrating dyes, industrial surfactants, and biocides [14]. The antibacterial activities of the R groups can also be affected by their length [15]. Typically, the strongest antibacterial action occurs in the methyl group lengths of C12–C16. QACs disrupt bacterial cytoplasmic membrane integrity and ionic stability. In addition to DNA binding, QACs also interact with intracellular targets.

At concentrations ranging from 0.5 to 5 mg/L, QACs exhibit algistatic, bacteriostatic, tuberculostatic, sporostatic, and fungistatic properties [14]. At concentrations ranging from 10 to 50 mg/L, the microbicidal properties of these substances were observed across the categories, with the efficacy varying based on the specific organism and formulation. A sequence of events describing the mechanism of action of QACs against microorganisms has been proposed to include: (i) adsorption and penetration of QACs into the cell wall; (ii) subsequent interaction of QACs with the cytoplasmic membrane, either through lipid or protein binding, leading to membrane disorganization; (iii) release of lower-weight intracellular components due to membrane permeability; (iv) degradation of proteins and nucleic acids within the cell; and (v) eventual lysis of the cell wall, facilitated by autolytic enzymes [16]. Different formulations of QACs have demonstrated efficacy against diverse microbial species [14]. A range of biocides have been prohibited for application to items intended for disposal through drainage systems. Therefore, it is imperative to prioritize chemicals that remain permissible and continue to be utilized in substantial quantities for research [17]. According to the United States Food and Drug Administration (FDA), benzalkonium chloride is classified as a Category III antiseptic active component. Ingredients were classified as Category III because there was insufficient information to determine their safety and effectiveness, necessitating further study. Two examples of antimicrobial agents commonly used in soaps are benzalkonium chloride, which is often found in bar soaps, and benzethonium chloride, which is commonly used in liquid-hand soaps. Benzethonium chloride, benzalkonium chloride, and cetylpyridinium chloride are chemical compounds with unique functions in the composition of DAs [7].

### 2.1. Benzalkonium Chloride

**Figure 1:**
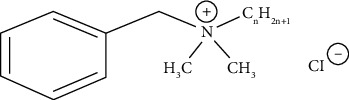
General chemical structure of benzalkonium chloride [19].

Benzalkonium chloride (BAC; CAS 8001-54-5) (Figure 1) is a quaternary ammonium chemical that has several functions, including application as a biocide, cationic surfactant, and phase transition agent [18]. Furthermore, it serves as an antiseptic agent, employed for the preservation of pharmaceuticals, or as a disinfectant for cleaning agents. BAC consists of a blend of alkyl benzyl dimethyl ammonium chloride, wherein the alkyl chain exhibits varying numbers of carbon atoms, typically n-C12H25 (dodecyl), n-C14H29 (tetradecyl), and n-C16H33 (hexadecyl). The antimicrobial potency is directly proportional to the length of the alkyl chain. The C12-homolog demonstrates efficacy against fungal and mold species, whereas the C14 variant exhibits activity against Gram-positive bacteria, and the C16 variant targets Gram-negative bacteria. Owing to the ability of BAC to rupture the cell membranes of microbes, it is frequently included in antiseptic compositions and demonstrates efficacy against a diverse array of bacteria, fungi, and certain types of viruses at low concentrations. The introduction of novel coatings and disinfection formulations based on QACs has led to a rise in their overall usage. Consequently, there has been an accompanying spike in reservations regarding the potential environmental and health risks associated with QAC exposure.

### 2.2. Benzethonium Chloride

**Figure 2:**
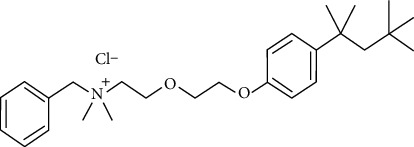
Chemical structure of benzethonium chloride [21].

Benzethonium (CAS 121-54-0) (Figure 2) is a synthetic quaternary ammonium salt that exhibits surfactant characteristics, possesses antiseptic properties, and demonstrates a wide range of antibacterial actions. Benzethonium chloride is primarily applied in its salt form as a skin disinfectant. It is also present in cosmetic products and personal care items, including mouthwashes and ointments, and is designed to alleviate itching. The efficacy of this substance in facilitating antibacterial activity against bacteria, fungi, molds, and viruses has been previously demonstrated. The concentrations often employed for this purpose range from 0.1% to 0.2%. These concentrations have been determined to be appropriate for use, as stated by the U.S. FDA [22]. Benzethonium is a member of the cationic detergent group that disrupts lipid bilayers. It is widely used because of its broad-spectrum antimicrobial activity against bacteria, viruses, and fungi [23]. Benzethonium chloride in antiseptic solutions kills bacteria by interfering with the integrity of microbial cell membranes. This leads to the leakage of cell contents and eventual cell death [24]. Benzethonium chloride is an active component of certain hard-surface disinfection solutions listed by the Environmental Protection Agency (EPA).

### 2.3. Cetylpyridinium Chloride

**Figure 3:**
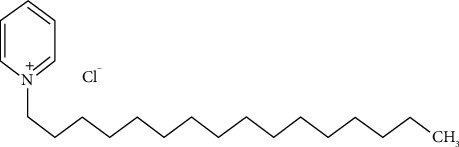
Chemical structure of cetylpyridinium chloride [25].

Cetylpyridinium chloride (CAS 123-03-5) (Figure 3) is another QAC used in various oral hygiene products and disinfectants [26]. It acts as an antiseptic by disrupting the cell membranes of microorganisms, resulting in inactivation. Cetylpyridinium chloride is utilized in oral hygiene solutions to effectively manage bacterial proliferation, mitigate plaque production, and deter the occurrence of halitosis [27]. The function of this entity is vital for the preservation of oral health and prevention of oral infections. Like other QACs, the excessive and prolonged use of goods containing cetylpyridinium chloride might result in the emergence of bacterial resistance. Strains that exhibit resistance have the potential to have diminished susceptibility to other antimicrobial agents, such as antibiotics, because of the common mechanisms of resistance.

Benzethonium chloride, benzalkonium chloride, and cetylpyridinium chloride are valuable components for the formulation of antiseptics and disinfectants. Their distinct mechanisms of action, ranging from cell membrane disruption to inhibition of bacterial growth, contribute to their effectiveness in preventing infections, controlling microbial growth, and maintaining public health in various settings.

## 3. Fate and Impact of QACs on the Environment

The distribution and incidence of QACs in the environment are influenced by their inherent properties. The presence of smaller QACs in wastewater and water can be attributed to their low solubility and hydrophobicity [28]. The permanent positive charges of QACs facilitate sorption to negatively charged solids, particularly phyllosilicate clay minerals, which are commonly found in soils and sediments. The vapor pressures of QACs restrict volatilization following indoor applications, thereby contributing to their persistence on indoor surfaces. The persistence of QACs is determined by their architecture and prevailing environmental conditions. Despite the potential for aerobic degradation and passive photolysis of QACs, their relevance is constrained by their pronounced affinity for the adsorption of particles and chemicals. [28, 29]. The detailed occurrences of the selected QACs in the environment are highlighted in Table 1.

QACs are ubiquitous in the environment globally, and their presence is not limited to household wastewater and sludge but also extends to treated effluent, surface water, and sediment [14]. Despite undergoing conventional wastewater treatment, QACs are not eliminated from the treated effluents. QACs are partially removed through processes such as sorption of biosolids and biological biodegradation [24]. In addition, QACs can still be found in the surface waters that receive them, especially at higher concentrations further downstream from the points where municipal wastewater treatment plant (WWTP) effluents and effluents from hospitals and industries such as laundry and food processing are released. Furthermore, the presence of nonlinear isotherms during adsorption suggests that QACs have a greater affinity for smaller amounts of organic particulates.

The notable levels of QAC present in the environment can be linked to the manufacturing of chemicals. As a result, with the rising global demand for QACs, these compounds are progressively being introduced into the environment via point source pollution, the land application of biosolids, or the discharge of treated municipal and industrial effluents [36]. Consequently, the occurrence of BACs in WWTPs, surface waters, and sediments could negatively impact various forms of life due to their toxic characteristics. Due to their widespread application, QACs are commonly found in influent, effluent, and receiving water bodies at levels that can be toxic. Approximately 25% of QACs ultimately find their way into the surrounding environment. Ventullo and Larson [37] found that QAC levels below 1 mg/L might induce ecologically important reactions.

The presence of QACs in ambient environments can be attributed to their extensive production and widespread usage. Hence, as the worldwide demand for QACs continues to increase, these substances will progressively infiltrate the environment via point-source pollution, application of biosolids to land, or discharge of treated municipal and industrial effluents. Consideration of chemical persistence is important when determining the prioritization of substances that are of interest. Existing regulations commonly establish criteria for determining the long-term stability of chemicals by considering their half-lives in various environmental nexuses. These criteria generally establish boundaries in the range of months, assuming situations that are favorable for biodegradation, such as high oxygen levels and the presence of acclimated bacteria. However, it is important to note that these standards can differ significantly and may not accurately represent the ambient conditions relevant to QACs [38]. Although they can degrade in the environment, they are classified as pseudo-persistent compounds. Furthermore, the sorption affinity of QACs in nonaqueous phases coupled with their persistent charges has the potential to hinder the biodegradation rate. Consequently, this can result in an extended half-life (spanning years) when QACs are sorbed into soil and sediment, thereby satisfying the persistence criterion [39].

In aquatic environments, three main processes facilitate the attenuation of QAC: photolysis, biodegradation, and the sorption of suspended particles, which ultimately results in settling. The quality assurance of QACs chemicals is generally recognized for its stability and relatively moderate degradation rates, influenced by processes such as hydrolysis, photolysis, and microbial activity. Research on the biodegradation of (QACs) has primarily utilized activated sludge or enrichment cultures. Notable data indicate that aquatic microbes can degrade and mineralize alkyltrimethyl ammonium compounds (ATMACs) and BACs to carbon dioxide within a period ranging from three days to two weeks [40], as described by [40, 41]. The biotransformation pathways of various QACs require complex bacterial consortia, rather than single species, to govern the fate of BAC in both natural and artificial systems. QAC concentrations were detected in both surface water and wastewater effluent [44]. These concentrations were measured to be as low as 0.002 μg/L and as high as 60 μg/L, respectively [45, 46]. Additionally, QACs in influent wastewater were significantly more concentrated, with concentrations up to 170 μg/L, representing a multiple-fold increase in concentration.

The utilization of nonpharmaceutical methods during and after the SARS-CoV-2 (COVID-19) pandemic has led to a significant increase in advertising and use of disinfectants containing QACs in healthcare and community environments. Disinfectants containing QACs account for more than 50% of items designated by the United States EPA and Health Canada as effective against SARS-CoV-2 [47, 48]. The recent COVID-19 pandemic has resulted in a significant increase in the use and environmental release of QACs [49]. The concentrations of QACs in aqueous environments range from 10^−3^ to 10^−1^ mg/L, with levels in wastewater being 10 times higher [28, 29, 50]. Consequently, it is imperative to recognize QACs as an emerging chemical class of concern.

However, there is a growing apprehension that extensive utilization of QACs might facilitate the development of bacterial resistance to QACs or trigger the development of resistance to antibiotics. Improper disinfectant use, a significant increase in QACs in untreated wastewater in specific regions, significantly elevated QAC levels in household dust compared to prepandemic levels, and the discovery of QACs in blood and breast milk samples from individuals during the pandemic have raised concerns about their safety [51]. The toxicity of QACs increases with prolonged exposure, as well as with physiological characteristics that provide resilience to QACs [52]. A study conducted by Alaa et al. [53] in Iran aimed to investigate the correlation between antibiotic resistance in bacteria and resistance to QACs in maternity wards. *Enterobacter cloacae*, *Escherichia coli*, certain serotypes of *Staphylococcus hominins*, and *Aeromonas hydrophila* were isolated from maternity wards and demonstrated resistance to disinfectants containing QACs.

Baseline activation of chromosomally encoded nonspecific efflux pumps confers innate resistance to QAC. The efflux determinants typically provide resistance against various agents, including antibiotics from the last resort class, and play physiological roles, such as conferring resistance against naturally occurring substances produced by the host, including bile hormones and host defense molecules [53]. Tolerance to QACs can be altered by sublethal exposure, which leads to relative changes. This is accomplished by temporarily modifying the density and nature of porins in the outermost cell membrane [53, 55]. Additionally, hyperexpression of efflux pumps is regulated in response to oxygen deprivation. Tolerance can also be affected by self-induced mutations. The acquisition of QAC-specific efflux pumps is facilitated by mobile recombinant genetic materials, such as plasmids and integrons [56]. Numerous aerobic and facultative microorganisms develop resistance to QACs by modifying their outer cell layer, specifically by altering the mix of fatty acids, phospholipids, and polysaccharides found within the cell membrane [57]. After these changes, the cellular composition becomes more negatively charged and hydrophobic, which makes it more difficult for the QAC to pass through the cell membranes. These effects could contribute to the colonization and long-term survival of bacteria within the host.

Co-resistance refers to the simultaneous selection of multiple genetic elements that confer resistance, including plasmids, transposons, and integrons, which are transferred concurrently [58]. Genes were individually expressed to determine their resistance. Cross-resistance occurs when a microorganism exhibits resistance to multiple unrelated compounds via a common mechanism such as employing the same efflux pump to counteract different antibiotics. Cross-resistance to QAC, for example, benzalkonium chloride, has also been reported. Merchel Piovesan Pereira et al. [23] subjected 40 *E. coli* strains to subinhibitory concentrations of 10 commonly utilized DAs. Seventeen strains showed cross-resistance to ampicillin, chloramphenicol, and norfloxacin. These results indicated that BAC induced cross-resistance. Membrane-related mutations are prevalent, and most strains exhibit enhanced biofilm-forming abilities [59].

In a separate investigation, *E. coli* strains were obtained from pigs, pig carcasses, and pork and were then exposed to BAC and other disinfectants. The strains were subsequently tested for the presence of eight antibiotics. BAC induced cross-resistance to at least three of the eight antibiotics. The efflux pump inhibitor (EPI) phenylalaninearginine-naphthylamide (PAbN) showed resistance to chloramphenicol and trimethoprim, while maintaining resistance to other antibiotics [60].

Recent findings during the COVID-19 pandemic highlighted the ability of *Listeria monocytogenes* to adapt to biocides, particularly QACs, and suggested a potential link between this adaptation and the selection of resistance to critical antibiotics like ciprofloxacin. The data indicate a potential association between the widespread application of QACs from production to consumption and the emergence of biocide and antibiotic resistance in pathogenic bacteria, including *Listeria monocytogenes* [61].

Jia et al. [48] investigated the molecular mechanisms of antibiotic resistance induced by mono- and twin-chained QACs, revealing a range of mutations associated with QACs. These mutations involve negative regulators (acrR, marR, soxR, and crp), outer membrane proteins and transporters (mipA and sbmA), and RNA polymerase (rpoB and rpoC), which may contribute to elevated multidrug resistance (MDR). Upon the removal of QACs stress, the phenotypic resistance caused by subinhibitory concentrations of QACs was found to be reversible, in contrast to the irreversible nature of resistance induced by inhibitory concentrations of QACs. The evolution and potential spread of antibiotic resistance due to rising environmental levels of (QACs), particularly dodecyl dimethyl ammonium chloride (DDAC), deserves critical attention.

### 3.1. Adaptation, Tolerance, and Resistance Mechanisms of Bacterial Pathogens to QAC (*Pseudomonas aeruginosa, Enterococcus* Species, and *Acinetobacter baumannii*)

*Enterococcus faecium, Staphylococcus aureus, Klebsiella pneumoniae, Acinetobacter baumannii, Pseudomonas aeruginosa*, and *Enterobacter* spp. (ESKAPE) pathogens frequently exhibit MDR in both developed and developing countries, indicating their ability to withstand more than three classes of antibiotics, which significantly contributes to the increasing prevalence of antibiotic resistance [62]. ESKAPE pathogens pose a significant global health challenge, complicating treatment options for severe infections, escalating the burden of disease, raising mortality rates due to treatment failures, impacting the achievement of sustainable development goals, and necessitating a unified global approach for surveillance of AMR [63].

*Acinetobacter baumannii, Pseudomonas aeruginosa*, and *Enterococcus* spp. have become prominent nosocomial pathogens, primarily owing to their remarkable ability to rapidly acquire resistance to a wide range of antimicrobial agents, and their ability to persist in the environment, humans, and animals [64–67].

### 3.2. *Pseudomonas aeruginosa*

*Pseudomonas* is a diverse genus of bacteria that is widely distributed in environmental niches, including water, wastewater, and plant surfaces. Furthermore, *Pseudomonas aeruginosa* is a significant nosocomial bacterium found in tap, recreational, and surface water and has been implicated in several infectious outbreaks in immunocompromised individuals [68, 69]. It is a widely distributed bacterium that thrives in many ecological environments because of its exceptional capacity to adapt, which is supported by its genome that is flexible and highly adaptable to different diets [70]. The remarkable ability of the bacterium to evade antimicrobial drugs is attributed to a wide array of innate and acquired resistance mechanisms within its genetic diversity.

These populations have been shown to adapt to subinhibitory concentrations by changing the structure of cell membranes, increasing the expression of efflux pumps, and accelerating biofilm growth and the capacity for persistent infections in both animal and human hosts [28, 71]. These attributes render this species especially appropriate for studying the impact of consistent subinhibitory levels of QACs. Lower concentrations of QACs can selectively affect the types of microorganisms that live in an area, possibly leading to the emergence of QAC-resistant populations. The primary cause of aerobic biodegradation of QACs has been identified as a bacterial species belonging to the *Pseudomonas* genus. The aerobic degradation of QACs is contingent on the presence of monooxygenases, dioxygenases, amine dehydrogenases, and/or amine oxidases [36]. The enzyme amine oxidase can significantly decrease BAC toxicity to bacteria by a factor of multiple hundred-fold. Approximately a decade ago, a gene cluster that encodes transporters, namely integrase and dioxygenase, was shown to be crucial for the biotransformation of BAC in aerobic environments [35]. The presence of multidrug efflux pump genes, including sugE, PmpM, mexAB-oprM, and mexEF-oprN, in the BAC-degrading community suggests that these efflux pumps and oxidase enzymes contribute to the enhancement of microbial resistance to QACs [72, 73].

### 3.3. *Enterococcus* Species

Among the more than 60 recognized *Enterococcus* species, *Enterococcus faecalis* and *Enterococcus faecium* are mostly associated with opportunistic human infections and are among the most common in the human gut microbiota [74]. It is important to examine how DAs affect the evolution of the *Enterococcus* microbiome, because this organism is one of the most common MDR healthcare-associated infections worldwide [75]. Inadequate cleaning during disinfection, improper biocide application, or the use of adulterated DAs in veterinary, food chain (e.g., food industry and farms), or human domestic contexts may lead to the use of QACs at subinhibitory concentrations in these specific contexts. Diverse microbiomes frequently encounter varying concentrations of QACs, potentially leading to alterations in the cellular composition of pathogens and fostering the selection of populations with reduced susceptibility to QACs and other antimicrobial agents (biocides or antibiotics) via co- or cross-selection mechanisms. The diminished sensitivity of *Enterococcus* species to QAC after adaptation may be attributed to several factors, including alterations in membrane fatty acid content, differential expression or mutations in efflux pumps, and stress responses. These findings reveal the complex bacterial adaptation mechanisms of QACs, potentially affecting disinfection strategies in healthcare. Understanding these processes may improve antimicrobial formulation and infection control protocols, thereby enhancing food and patient safety.

### 3.4. *Acinetobacter baumannii*

*Acinetobacter* is ubiquitous in nature and has been isolated from both clinical and environmental samples. MDR *Acinetobacter baumannii* has substantial clinical importance due to its role in hospital-associated nosocomial epidemics worldwide [76]. The potential impact of QAC and its derivatives on the spread of antimicrobial-resistant genes/bacteria via co-selection processes in manure and WWTPs, or in environments where *Acinetobacter baumannii* and QAC come into close contact [77]. High concentrations of BZK mostly cause membrane damage, although low concentrations have been demonstrated to disturb the balance of proteins in cells, ultimately resulting in the death of *A. baumannii* cells. The resistance and tolerance of *Acinetobacter baumannii* to QACs are frequently linked to mobile genetic elements (MGEs), which facilitate the extensive spread of AMR genes and bacteria. *A. baumannii's* array of multidrug efflux pumps contribute to its high disinfectant (chlorhexidine and benzalkonium chloride) tolerance, which gives it a unique capacity to persist for extended periods of time in hospital conditions [78, 79]. Currently, resistance to disinfectants is an adaptive advantage. However, many *A. baumannii* efflux pump genes have been found in all species or genera, suggesting that they have a common function in the past [80]. The arrays of bacterial pathogens exhibiting QAC resistance patterns are shown in Table 2.

## 4. Mechanism of QAC Resistance

When disinfectants are used incorrectly or bacteria are exposed to doses below what is needed to kill them, some types of bacteria may be able to survive, become less sensitive, and develop resistance. QAC-resistant microorganisms are frequently obtained from sewage or activated sludge due to the discharge of QACs into the environment via runoff [36, 97, 98]. This phenomenon gives rise to concentration gradients in QACs, facilitating microbial adaptability to subinhibitory concentrations of disinfectants [100]. Antibiotic resistance has received increased attention since the 1960s. However, it is important to note that the escalation of resistance to disinfectants and antibiotics poses a substantial risk to biosafety and human health. In addition to responses to exposure to antibiotics, bacteria could adapt to changes in their ambient environment, including exposure to antimicrobial chemicals, and thereby exhibit resistance to disinfectants. Because of this, changes have been seen in the membranes of cells, the appearance of mutant enzymes with new functions and metabolic pathways, and the activation of more resistance genes, such as those linked to horizontal gene transfer (HGT), biofilm formation, different efflux pumps, and biotransformation.

### 4.1. HGT of QAC and Antibiotic Genes

Drug-susceptible bacteria can acquire antibiotic resistance, primarily through genetic mutations or gene transfer, with HGT being the predominant mechanism. Mutations occur randomly and are typically spread through vertical inheritance; however, HGT is more concerning because it allows for the phylogenetic jumps of genes [7]. This suggests that the majority of the resistance genes found in microbiomes are part of the intrinsic resistome. The primary concern regarding HGT mechanisms in disinfectants and the spread of antiseptic resistance is conjugation. QACs facilitate the transfer of genetic material between bacteria via direct cell-to-cell contact [7]. Hu et al. [50] systematically investigated the effects of various QAC with differing structures and carbon chain homology on the conjugative transfer of antibiotic resistance genes (ARGs). Their findings indicated that most QACs at environmental concentrations could facilitate the horizontal transfer of ARGs from *E. coli* DH5*α* to *E. coli* MG1655 and *S. sonnei* through various mechanisms. The reported concentrations of QACs in aqueous environments range from 10^−3^ to 10^−1^ mg/L. Without accounting for the synergistic or antagonistic effects of other environmental factors, it is important to highlight the potential risks of QACs facilitating the horizontal transfer of ARGs in the environment. *S. sonnei* is a pathogen that affects the human intestine, with its infection rate rising annually. This study demonstrated that *S. sonnei* can obtain ARGs from environmental bacteria through HGT, with exposure to QACs that enhance the transfer rate.

### 4.2. Efflux Pumps as a QAC Resistance Mechanism

**Figure 4:**
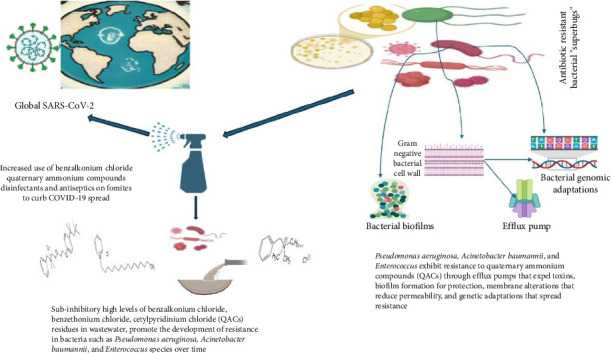
Collectively, this interconnected narrative illustrates the progression from increased disinfectant use due to SAR-CoV-2 pandemic to environmental pollution and the subsequent rise of antimicrobial-resistant pathogens.

Efflux pumps are the basic mechanisms of resistance to DAs. The concentrations of antimicrobial agents within cells are reduced by actively transporting agents out of the cells [101], as described by [99]. Efflux pumps can exhibit either particularity towards a substrate or possess a broad spectrum of substrates, as seen by MDR pumps. Efflux-mediated intrinsic resistance is a mechanism that enables tolerance to QACs in amounts that are considered environmentally harmful, as shown in Figure 4. Efflux-mediated QAC resistance has received attention because of its genetic basis, ability to create simultaneous resistance to antibiotics, and potential for HGT across different microbial species [103]. Proteins facilitate active expulsion of antimicrobials within bacterial cells. Efflux systems can actively transport a diverse array of chemically and structurally dissimilar substances, such as QACs, using energy- or proton-dependent mechanisms, while maintaining the integrity of transported chemicals [104, 105]. There are various categories of multidrug efflux systems, including (i) resistance-nodulation-division (RND), (ii) major facilitator superfamily (MFS), (iii) adenosine triphosphate (ATP)-binding cassette (ABC) family, (iv) small multidrug resistance (SMR) family, (v) multidrug and toxic compound extrusion (MATE) family, and (vi) proteobacterial antimicrobial compound efflux (PACE) [104, 106] MDR efflux systems, including the MFS, ATP-ABC, and SMR systems, are frequently observed in Gram-positive bacteria. Conversely, the RND, NorA, and QacA/A families of efflux pumps are more commonly associated with Gram-negative bacteria. The MATE family pump, known as PmPM, is present in several species of bacterial, including *P. aeruginosa, Acinetobacter baumannii, Haemophilus influenzae, Clostridium difficile, Vibrio parahaemolyticus*, *Bacteroides thetaiotaomicron*, and *Staphylococcus aureus* [107]. The upregulation of efflux pumps results in a 2- to 8-fold increase in resistance to QAC and other associated substrates transported by the pump. ABC transporters belong to the basic family class, whereas the remaining families are regarded as the advanced family class.

RND efflux pumps are mostly found in Gram-negative bacteria. They have three parts: a cytoplasmic membrane pump, a periplasmic protein, and an outer membrane protein channel. The AcrAB-TolC system, which belongs to the RND family, is prevalent in Gram-negative bacteria, including *Escherichia coli*. This system has a broad substrate specificity, including dyes, fluoroquinolones, and BAC. Proportion of subpopulations persisting significantly decreased by 1000-fold when the tolC gene was knocked out. This observation suggests that *TolC* facilitation of the efflux mechanism is crucial for the persistence of BAC tolerance in subpopulations. These results are in line with those of other studies that have found links between differences in the location of the AcrAB-TolC efflux pump, differences in tolC expression, and antibiotic resistance. The results of a recent study suggested that persistent cells exhibit greater tolerance to bactericidal antibiotics because of enhanced efflux mechanisms [100]. Further studies are required to validate this mechanism. This hypothesis posits that the random expression of the marRAB system, which serves as a regulator of acrAB and tolC51, can potentially contribute to the survival of artificial chromosomes in bacteria by inducing heterogeneous gene expression.

Resistance against ATMACs through efflux pumps might be attributed to two distinct processes: First, efflux pumps are overexpressed in response to exposure to high concentrations of QACs, which then results in resistance against QACs; Second, internally generated QAC stress can trigger overexpression of efflux pumps, resulting in resistance against QACs. Exposure to such stressors can activate a regulatory mechanism responsible for modulating the expression of efflux determinants. Alternatively, stress can cause one or more mutations in the genetic elements, which results in increased production of the efflux driver or makes its outflow more effective [108, 109]. In this context, genes encoding proteins associated with efflux pumps are often encoded within chromosomes or acquired through MGEs and exhibit activities against a diverse range of antimicrobial drugs. QAC resistance genes, such as class-1 integrons are frequently located in the same genetic elements as the antibiotic resistance genes. This colocalization potentially facilitates the emergence of co-resistance between QACs and antibiotics because they might possess comparable modes of action [52, 110]. Exposure to comparable sub-inhibitory concentrations of QACs or DAs can lead to the survival of potentially pathogenic microbes. Survival could result in the emergence of cross-resistance against medications that share structural or functional similarities, including antibiotics that are often used in medical interventions [111]. This phenomenon presents a plausible mechanism for producing antibiotic-resistant bacteria in the surroundings contaminated with QACs. It is possible for QAC efflux pump genes to be moved from one cell to another through conjugation, which can be accomplished by integrons, plasmids, transposons, and conjugative elements [112]. The qac gene provides an initial selection advantage by conferring resistance to DAs. Hence, the increasing use of QACs facilitates the fixation of additional genetic elements that are new and distinct. Integrons serve as reservoirs for resistance cassettes and can efficiently acquire supplementary resistance determinants through plasmids. The present vehicle technology demonstrates high efficiency in facilitating interspecies mobility. It is common for Gram-negative bacteria to become resistant to QACs by having qac genes, and for Gram-positive bacteria to become resistant by having class 1 integrons [113]. It is important to acknowledge that reduced sensitivity and sometimes resistance against QACs is facilitated by efflux pumps, among other possible mechanisms. Numerous studies have indicated that efflux pumps might facilitate biofilm formation either through direct or indirect mechanisms [114, 115]. Empirical and genetic evidence supports the idea that QAC environmental residues might potentially have a significant impact on the evolution of AMR, and the transfer of genes linked to AMR [98]. Because of its extensive array of efflux pumps, which can expel many antimicrobial agents, including QACs and antibiotics, the genus *Pseudomonas* has significant relevance in the context of AMR, owing to its extensive distribution in many environments. Its inclusion as one of the most notorious drug-resistant bacteria poses significant challenges in the treatment of bacterial infections [28, 116]. QACs facilitated the transfer of RP4-resistant plasmids into *E. coli* at low doses. The quantities of QACs that maximized the enhancement of trans-conjugate efficiency were comparable to those observed in the environment. This indicates that the presence of residual QACs in the environment might worsen environmentally induced resistance of bacteria to antimicrobials and/or antibiotics [117].

### 4.3. Biofilm Formation as a QAC-Resistant Mechanism

Compounds such as benzalkonium chloride and chlorhexidine can potentially induce biofilm development. Biofilms, which are intricate assemblies of microorganisms surrounded by a protective extracellular matrix, serve as physical barriers that can confer protection against antimicrobial agents, such as antibiotics. It has been suggested that EPS matrices enhance recalcitrance by impeding the diffusion of disinfection solutes into biofilms. This hindrance can occur through mechanisms such as size exclusion, interactions, and reactions with solutes [118]. Biofilm formation contributes to a decrease in the vulnerability of both Gram-positive and Gram-negative bacteria to disinfectants. Gram-negative bacteria have a greater propensity to acquire resistance against QACs because of the composition of their cell walls [119]. Furthermore, the extracellular matrix contains other constituents, including enzymes, that can contribute to the detoxification of harmful substances. For example, *P. aeruginosa* can withstand exposure to disinfectants through the formation of persistent cells, effectively hindering the manufacture of antibiotic targets [120, 121]. According to Qin et al. [122], the presence of molecules derived from persistent cells can sustain their viability and replenish biofilms. In the absence of disinfectants, bacteria regain growth and viability, resulting in the development of chronic infections. The effectiveness of disinfectants against biofilms is significantly lower than that of planktonic organisms [123].

The resistance of biofilms to disinfectants is contingent on factors such as the efficacy of disinfectants, temperature, and surface characteristics [125]. In addition, bacterial biofilms formed within wastewater treatment systems can function as reservoirs of AMR. Biofilms provide a shield against antimicrobial agents, allowing bacteria to survive and potentially exchange resistance genes within the biofilm community [10]. The coexistence of various microorganisms within the surrounding ecosystem may potentially contribute to increased survival when exposed to disinfectant-induced stress as shown in Figure 4. For example, *Listeria monocytogenes* monocytes have been shown to protect against Pseudomonas putida when exposed to low levels of unclassified BAC [126]. The treatment selectively targeted and eradicated *L. monocytogenes*. Another discovery was that the addition of *Pseudomonas fluorescens* to dual-species biofilms made *Salmonella enterica* more resistant to a group of unknown QACs [121, 127].

### 4.4. Biotransformation as a QAC-Resistance Mechanism

Most types of antibiotics can be broken down by photo- or hydrolytic activity in the environment because of the way their molecules are structured and the physical and chemical properties they have [124]. The initial steps of QAC biodegradation are a multistep process conducted by microorganisms, which is similar to the degradation of antibiotics. Metabolism requires the recognition of the molecule and efficient binding between enzymes and substrates [129]. This phenomenon is contingent on the arrangement of the molecule and its physicochemical characteristics. The metabolic processes of QACs are influenced by their structural compositions. Consequently, the presence of hydrophobic alkyl chains has been shown to significantly affect the biodegradation and toxicity of QACs. Specifically, owing to their lower solubility, the elongation of alkyl chains has been shown to reduce biodegradability [130, 131]. Hence, reduced solubility restricts the availability of microbial species. DAs might have less of an effect on the ecosystem if there is a diverse community of microbes that include species with moderate to high tolerance and the ability to break down antimicrobial compounds. Because biotransformation reduces the concentrations of antimicrobials, exposure-susceptible species may reach subinhibitory concentrations. This could facilitate the development of resistance. Previous studies have demonstrated that certain bacteria possess the ability to metabolize disinfectants, such as QACs. These microbes respond to antimicrobial agents and may potentially acquire resistance through several metabolic pathways [132, 133].

Biotransformation is likely to be the primary method for removing disinfectants such as QACs from wastewater treatment facilities. When a community of microbes is exposed to QACs, the genera that are naturally resistant or that can biotransform QACs are given more attention [134]. For instance, a previous study reported that after exposure to BAC, microbial communities exhibited a decrease in activity and were composed of *Pseudomonas* sp., as described by [99, 131]. *P. nitroreducens*, *P. aeruginosa*, and *P. putida* were the *Pseudomonas* species that were most often seen after being exposed to BAC in the special enrichment medium. Additional taxa, including *Citrobacter* sp., *Klebsiella* sp., *Salmonella* sp., *Enterobacter* sp., and *Achromobacter*, have been identified [36]. Relative to enhanced microbial communities, bacterial artificial chromosomes (BACs) undergo degradation via dealkylation, resulting in the formation of BDMA. This is followed by successive demethylation, resulting in the production of dimethylamine and methylamine [99]. With the development of resistance, microbes may acquire the capacity to use antimicrobials as a viable source of nutrition or energy. These microbes, which are related to *P. nitroreducens* and *P. putida*, have been linked to the process of assimilation and degradation of BAC [135]. This phenomenon not only provides advantages to the destruction of microorganisms but might also offer protection to other members of the community because of the influence effect.

The continuous emergence of QAC resistance in the environment necessitates urgent development of next-generation disinfectants for human health. It is essential to balance antibiotic efficacy with environmental factors in order to successfully combat the emergence of bacterial resistance [135]. Recent research has shown that biscationic quaternary phosphonium compounds (bis-cationic QPCs) are very effective against both Gram-positive and Gram-negative pathogenic bacterial strains that can acquire QAC resistance mechanisms [135].

## 5. Conclusions

QACs are becoming more popular as preferred agents for chemical disinfection of microorganisms in healthcare, household, and industrial settings. Improper utilization of these substances has the potential to exert selection pressure, contributing to the development of resistance, including AMR, which is currently a widespread phenomenon worldwide. It is very important to make sure that policies are put in place to encourage responsible and smart use, as this will help lower the risks that come with the spread of antimicrobial-resistant strains in the years after COVID. Policymakers should mandate the disclosure of QACs and their possible effects to encourage responsible usage by consumers, while fostering stewardship among producers. In addition, the use of environmentally sustainable QACs or alternative chemicals, together with improved practices for the treatment and control of wastewater containing QACs, presents encouraging avenues for mitigating or eradicating the discharge of QACs into progressively strained ecosystems. Moreover, it is important to enhance monitoring systems to effectively monitor the establishment and dissemination of AMR, which is crucial for the development of successful intervention approaches. Moreover, DAs and antibiotics play a crucial role in controlling infections caused by MDR bacteria. Therefore, the research community must continue to support studies that generate practical data to guide the development of policies. This can be achieved by offering incentives for research and development, which in turn can facilitate the development of novel antimicrobial drugs that specifically combat resistant infections. Nevertheless, despite some findings from experiments conducted in controlled laboratory environments, there is a dearth of definitive data indicating that QACs are directly responsible for the extensive proliferation of AMR in real-life scenarios. Further research is required to investigate the evolutionary patterns of pathogens that exhibit tolerance and resistance to QACs and antibiotics in real-world environments. Therefore, a more comprehensive understanding of the potential impact of QAC use on the promotion of clinically significant resistance is required.

## Figures and Tables

**Table 1:** Occurrence of QACs in wastewater, surface water and wastewater treatment plants.

QACs	Continent	Country(s)	Sources	Concentration μg/L	References
C_12_-benzalkonium chloride	Europe	Austria	Wastewater treatment plants (WWTPs)	170 μg/L	[30, 31]
	Europe	Germany	Wastewater	7.7 μg/L	[32]
	North America	USA	Surface water downstream from WWTPs	2.7–5.8 μg/L	[33]
	Africa	South Africa	WWTPs	7.805 μg/L	[34]

BAC C-14	Europe	Austria	WWTPs	110 μg/L	[31]
	North America	USA	Surface water downstream from WWTPs	6.3–36.6 μg/L	[33]

Benzalkonium chlorides	Southeast Europe and West Asia	Turkey	Surface water and wastewater	40 and 500 μg/L	[35]

BAC C12, BAC C14 and DADMAC C10	Europe	Austria	Surface water	1.9 μg/L	[29]

**Table 2:** Reported cases of bacterial pathogens resistant to quaternary ammonium compounds.

QAC resistant pathogens	Continent	Country	Sources	References
*Acinetobacter bohemicus strain*	Europe	Germany	Pig manure	[76]
ESBL-producing *Escherichia coli*	Europe	Central Slovenia	Human (lower respiratory infection)	[81]
*Escherichia coli*	North America	United States of America	Retailed meat	[12]
*Enterobacter cloacae*	Europe	Norway	Fish farm	[82]
*Escherichia coli*	Africa	Egypt	Chicken	[83]
*Escherichia coli*	Africa	Egypt	Diseased broilers and environmental sources	[84]
*Acinetobacter baumannii*	North America	United States of America	Clinical isolates	[85]
*Acinetobacter baumannii*	Asia	Malaysia	Admitted patients	[86]
*Klebsiella pneumoniae*	Asia	Saudi Arabia	Clinical specimen	[87]
*Pseudomonas aeruginosa*	Asia	Saudi Arabia	Clinical specimen	[87]
*Acinetobacter baumannii*	Asia	Saudi Arabia	Clinical specimen	[87]
*Acinetobacter* spp.	South America	Brazil	Food sample	[88]
*Acinetobacter baumannii*	South America	Venezuela	Patients with nosocomial infections	[89]
*Pseudomonas aeruginosa*	South America	Brazil	Clinical isolates	[90]
*Pseudomonas aeruginosa*	Australia	Australia	Ocular isolates	[91]
*Staphylococcus* spp.	Europe	Norway	Food industry	[90]
*Listeria monocytogenes*	Europe	Norway	Food industry	[92]
*staphylococci*	Europe	Norway	Clinical strains	[93]
*Listeria monocytogenes*	North America	United States of America	Fresh produce environment	[94]
*Lysinibacillus* sp.	Asia	China	Soil	[95]
*Bacillus aryabhattai*	Asia	China	Soil	[95]
*Bacillus cereus*	Asia	China	Soil	[95]
*Exiguobacterium acetylicum*	Asia	China	Soil	[95]
*Bacillus thuringiensis*	Asia	China	Soil	[95]
*Klebsiella pneumoniae*	Asia	China	Soil	[95]
*Enterobacter ludwigii*	Asia	China	Soil	[95]
*Salmonella enterica*	Asia	China	Retail meat, retail layer and human faeces	[96]

## Data Availability

No data are generated in this manuscript.

## Nomenclature

COVID-19: Coronavirus disease 2019
SARS-CoV-2: Severe acute respiratory syndrome coronavirus 2
QAC: Quaternary ammonium compounds
AMR: Antimicrobial resistance
FDA: Food and drug administration
BAC: Benzalkonium chloride
DNA: Deoxyribonucleic acid
EPA: Environmental protection agency
ADBAC: Alkyl dimethyl benzyl ammonium chloride
WWTP: Wastewater treatment plant
ATMAC: Alkyl trimethyl ammonium compounds
RND: Resistance-nodulation-division
MFS: Major facilitator superfamily
ATP: Adenosine triphosphate
ABC: ATP-binding cassette
SMR: Small multidrug resistance
MATE: Multidrug and toxic compound extrusion family
PACE: Proteobacterial antimicrobial compound efflux
MDR: Multiple-drug resistance
MFS: Major facilitator superfamily
AcrB: Acriflavine resistance B
DA: Disinfectant and antiseptic

## References

[1] Nanayakkara A. K., Boucher H. W., Fowler V. G., Jezek A., Outterson K., Greenberg D. E. (2021). Antibiotic Resistance in Patients with Cancer: Escalating Challenges and Paths Forward. *CA: A Cancer Journal for Clinicians*.

[2] Salam A., Al-Amin M., Salam M. T. (2023). Antimicrobial Resistance: a Growing Serious Threat for Global Public Health. *Healthcare*.

[3] Lomazzi M., Moore M., Johnson A., Balasegaram M., Borisch B. (2019). Antimicrobial Resistance–Moving Forward?. *BMC Public Health*.

[4] Chinemerem Nwobodo D., Ugwu M. C., Oliseloke Anie C. (2022). Antibiotic Resistance: The Challenges and Some Emerging Strategies for Tackling a Global Menace. *Journal of Clinical Laboratory Analysis*.

[5] World Health Organization (2022). Antimicrobial Resistance Surveillance in Europe 2022–2020 Data.

[6] Rahman M., Alam Tumpa M. A., Zehravi M. (2022). An Overview of Antimicrobial Stewardship Optimization: The Use of Antibiotics in Humans and Animals to Prevent Resistance. *Antibiotics*.

[7] Basiry D., Entezari Heravi N., Uluseker C., Kaster K. M., Kommedal R., Pala-Ozkok I. (2022). The Effect of Disinfectants and Antiseptics on Co- and Cross-Selection of Resistance to Antibiotics in Aquatic Environments and Wastewater Treatment Plants. *Frontiers in Microbiology*.

[8] Marteinson S. C., Lawrence M., Taranu Z. E. (2023). Increased Use of Sanitizers and Disinfectants during the COVID-19 Pandemic: Identification of Antimicrobial Chemicals and Considerations for Aquatic Environmental Contamination. *Environmental Reviews*.

[9] Mahoney A. R., Safaee M. M., Wuest W. M., Furst A. L. (2021). The Silent Pandemic: Emergent Antibiotic Resistances Following the Global Response to SARS-CoV-2. *iScience*.

[10] Boyce J. M. (2023). Quaternary Ammonium Disinfectants and Antiseptics: Tolerance, Resistance and Potential Impact on Antibiotic Resistance. *Antimicrobial Resistance and Infection Control*.

[11] Krüger M., Basiouni S., Eder I., Rodloff A. (2021). Susceptibility of Extended-Spectrum SS-Lactamase (ESBL)-producing *Enterobacteriaceae* to Roundup.

[12] Zou L., Meng J., McDermott P. F. (2014). Presence of Disinfectant Resistance Genes in *Escherichia coli* Isolated from Retail Meats in the USA. *Journal of Antimicrobial Chemotherapy*.

[13] Rutala W. A., Weber D. J. (2015). Disinfection, Sterilization, and Control of Hospital Waste. *Mandell, Douglas, and Bennett’s principles and practice of infectious diseases*.

[14] Gerba C. P. (2015). Quaternary Ammonium Biocides: Efficacy in Application. *Applied and Environmental Microbiology*.

[15] Jennings M. C., Minbiole K. P., Wuest W. M. (2015). Quaternary Ammonium Compounds: an Antimicrobial Mainstay and Platform for Innovation to Address Bacterial Resistance. *ACS Infectious Diseases*.

[16] McDonnell G. E. (2007). Mechanisms of Prion Resistance in Antisepsis, Disinfection, and Sterilization.

[17] Hhs (2016). Safety and Effectiveness of Consumer Antiseptics; Topical Antimicrobial Drug Products for Over-the-counter Human Use. Final Rule. *Federal Register*.

[18] Lackner M., Guggenbichler J. P. (2013). Antimicrobial Surfaces. *Ullmann’s Encyclopedia of Industrial Chemistry*.

[19] Benzalkonium Chloride (2025).

[20] Parthiban D., Karunakaran R. (2018). Benzethonium Chloride Catalyzed One Pot Synthesis of 2,4,5-Trisubstituted Imidazoles and 1,2,4,5-Tetrasubstituted Imidazoles in Aqueous Ethanol as A Green Solvent. *Oriental Journal of Chemistry*.

[21] Okeke C. A., Khanna R., Ehrlich A. (2023). Quaternary Ammonium Compounds and Contact Dermatitis: A Review and Considerations during the COVID-19 Pandemic. *Clinical, Cosmetic and Investigational Dermatology*.

[22] Merchel Piovesan Pereira B., Tagkopoulos I. (2019). Benzalkonium Chlorides Uses, Regulatory Status, and Microbial Resistance. *Applied and Environmental Microbiology*.

[23] Hora P., Pati S. G., McNamara P., Arnold W. A. (2020). Increased Use of Quaternary Ammonium Compounds during the SARS-CoV-2 Pandemic and beyond: Consideration of Environmental Implications. *Environmental Science and Technology Letters*.

[24] Zarei A. R., Sadeghi H. B., Abedin S. (2013). Selective Cloud Point Extraction for the Spectrophotometric Determination of Cetylpyridinium Chloride in Pharmaceutical Formulations. *Iranian Journal of Pharmaceutical Research*.

[25] Mao X., Auer D. L., Buchalla W. (2020). Cetylpyridinium Chloride: Mechanism of Action, Antimicrobial Efficacy in Biofilms, and Potential Risks of Resistance. *Antimicrobial Agents and Chemotherapy*.

[26] Liu J., Ling J., Wu C. D. (2013). Cetylpyridinium Chloride Suppresses Gene Expression Associated with Halitosis. *Archives of Oral Biology*.

[27] Arnold W. A., Blum A., Branyan J. (2023). Quaternary Ammonium Compounds: A Chemical Class of Emerging Concern. *Environmental Science & Technology*.

[28] Mohapatra S., Yutao L., Goh S. G. (2023). Quaternary Ammonium Compounds of Emerging Concern: Classification, Occurrence, Fate, Toxicity and Antimicrobial Resistance. *Journal of Hazardous Materials*.

[29] Martínez-Carballo E., Sitka A., González-Barreiro C. (2007). Determination of Selected Quaternary Ammonium Compounds by Liquid Chromatography with Mass Spectrometry. Part I. Application to Surface, Waste and Indirect Discharge Water Samples in Austria. *Environmental Pollution*.

[30] Zhang C., Cui F., Zeng G. M. (2015). Quaternary Ammonium Compounds (QACs): a Review on Occurrence, Fate and Toxicity in the Environment. *Science of the Total Environment*.

[31] Wieck S., Olsson O., Kümmerer K. (2018). Not Only Biocidal Products: Washing and Cleaning Agents and Personal Care Products Can Act as Further Sources of Biocidal Active Substances in Wastewater. *Environment International*.

[32] Ferrer I., Furlong E. T. (2001). Identification of Alkyl Dimethylbenzylammonium Surfactants in Water Samples by Solid-phase Extraction Followed by Ion Trap LC/MS and LC/MS/MS. *Environmental Science & Technology*.

[33] Chukwu K. B., Abafe O. A., Amoako D. G., Essack S. Y., Abia A. L. K. (2023). Antibiotic, Heavy Metal, and Biocide Concentrations in a Wastewater Treatment Plant and its Receiving Water Body Exceed PNEC Limits: Potential for Antimicrobial Resistance Selective Pressure. *Antibiotics*.

[34] Ertekin E., Hatt J. K., Konstantinidis K. T., Tezel U. (2016). Similar Microbial Consortia and Genes Are Involved in the Biodegradation of Benzalkonium Chlorides in Different Environments. *Environmental Science and Technology*.

[35] Oh S., Kurt Z., Tsementzi D. (2014). Microbial Community Degradation of Widely Used Quaternary Ammonium Disinfectants. *Applied and Environmental Microbiology*.

[36] Ventullo R. M., Larson R. J. (1986). Adaptation of Aquatic Microbial Communities to Quaternary Ammonium Compounds. *Applied and Environmental Microbiology*.

[37] Oecd (2021). OECD Test Guidelines for Chemicals.

[38] Li Y., Zhou C., Wang S. (2019). Phytotoxicity and Oxidative Effects of Typical Quaternary Ammonium Compounds on Wheat (Triticum aestivum L.) Seedlings. *Environmental Science and Pollution Research*.

[39] Patrauchan M. A., Oriel P. J. (2003). Degradation of Benzyldimethylalkylammonium Chloride by *Aeromonas hydrophila* Sp. K. *Journal of Applied Microbiology*.

[40] Zheng C. W., Luo Y. H., Long X. (2023). The Structure of Biodegradable Surfactants Shaped the Microbial Community, Antimicrobial Resistance, and Potential for Horizontal Gene Transfer. *Water Research*.

[41] Khan A. H., Topp E., Scott A., Sumarah M., Macfie S. M., Ray M. B. (2015). Biodegradation of Benzalkonium Chlorides Singly and in Mixtures by a *Pseudomonas Sp.* Isolated from Returned Activated Sludge. *Journal of Hazardous Materials*.

[42] Pati S. G., Arnold W. A. (2020). Comprehensive Screening of Quaternary Ammonium Surfactants and Ionic Liquids in Wastewater Effluents and Lake Sediments. *Environmental Science: Processes & Impacts*.

[43] Kreuzinger N., Fuerhacker M., Scharf S., Uhl M., Gans O., Grillitsch B. (2007). Methodological Approach towards the Environmental Significance of Uncharacterized Substances—Quaternary Ammonium Compounds as an Example. *Desalination*.

[44] Östman M., Lindberg R. H., Fick J., Björn E., Tysklind M. (2017). Screening of Biocides, Metals and Antibiotics in Swedish Sewage Sludge and Wastewater. *Water Research*.

[45] Health Canada (2020). *Hard Surface Disinfectants and Hand Sanitizers: List of Hard-Surface Disinfectants for Use Against Coronavirus (COVID-19)*.

[46] Jia Y., Lu H., Zhu L. (2022). Molecular Mechanism of Antibiotic Resistance Induced by Mono-And Twin-Chained Quaternary Ammonium Compounds. *The Science of the Total Environment*.

[47] Chen B., Han J., Dai H., Jia P. (2021). Biocide-tolerance and Antibiotic-Resistance in Community Environments and Risk of Direct Transfers to Humans: Unintended Consequences of Community-wide Surface Disinfecting during COVID-19?. *Environmental Pollution*.

[48] Hu Q., Zhang L., Yang R., Tang J., Dong G. (2024). Quaternary Ammonium Biocides Promote Conjugative Transfer of Antibiotic Resistance Gene in Structure-And Species-dependent Manner. *Environment International*.

[49] Zheng G., Webster T. F., Salamova A. (2021). Quaternary Ammonium Compounds: Bioaccumulation Potentials in Humans and Levels in Blood before and during the Covid-19 Pandemic. *Environmental Science and Technology*.

[50] Hegstad K., Langsrud S., Lunestad B. T., Scheie A. A., Sunde M., Yazdankhah S. P. (2010). Does the Wide Use of Quaternary Ammonium Compounds Enhance the Selection and Spread of Antimicrobial Resistance and Thus Threaten Our Health?. *Microbial Drug Resistance*.

[51] Alaa M. A., Ayad A., M M. A. (2023). Investigating the Correlation between Antibiotics Resistance of Bacteria and Quaternary Ammonium Compounds (QACs) Resistance in Maternity Wards. *Migration Letters*.

[52] Knapp C. W., Keen P. L., Montforts M. H. M. M. (2013). *Antimicrobial Resistance in the Environment*.

[53] Vereshchagin A. N., Frolov N. A., Egorova K. S., Seitkalieva M. M., Ananikov V. P. (2021). Quaternary Ammonium Compounds (QACs) and Ionic Liquids (ILs) as Biocides: from Simple Antiseptics to Tunable Antimicrobials. *International Journal of Molecular Sciences*.

[54] Huang L., Wu C., Gao H. (2022). Bacterial Multidrug Efflux Pumps at the Frontline of Antimicrobial Resistance: An Overview. *Antibiotics*.

[55] Barroso I. L. (2017). Listeria Monocytogenes Biofilms Produced under Nutrient Scarcity and Cold Stress: Disinfectant Susceptibility of Persistent Strains Collected from the Meat Industry in Spain (Doctoral Dissertation).

[56] Murray L. M., Hayes A., Snape J., Kasprzyk-Hordern B., Gaze W. H., Murray A. K. (2024). Co-selection for Antibiotic Resistance by Environmental Contaminants. *Npj Antimicrobials and Resistance*.

[57] Merchel Piovesan Pereira B., Wang X., Tagkopoulos I. (2021). Biocide-induced Emergence of Antibiotic Resistance in *Escherichia coli*. *Frontiers in Microbiology*.

[58] Puangseree J., Jeamsripong S., Prathan R., Pungpian C., Chuanchuen R. (2021). Resistance to Widely Used Disinfectants and Heavy Metals and Cross Resistance to Antibiotics in *Escherichia coli* Isolated from Pigs, Pork and Pig Carcass. *Food Control*.

[59] Guérin A., Bridier A., Le Grandois P. (2021). Exposure to Quaternary Ammonium Compounds Selects Resistance to Ciprofloxacin in *Listeria* Monocytogenes. *Pathogens*.

[60] Augustine F. F., Mgaya X. M., Yahya S. A., Niccodem E. M., Matee M. I. (2023). An Alarming Prevalence of Multidrug-Resistant (MDR) ESKAPE Pathogens and Other Drug-Resistant Bacteria Isolated from Patients with Bloodstream Infections Hospitalized at Muhimbili National Hospital in Dar Es Salaam, Tanzania. *German Journal Microbiology*.

[61] De Oliveira D. M., Forde B. M., Kidd T. J. (2020). Antimicrobial Resistance in ESKAPE Pathogens. *Clinical Microbiology Reviews*.

[62] Mellace M., Ceniti C., Paonessa M., Procopio A. C., Tilocca B. (2024). Multidrug Resistant *Acinetobacter baumannii*: an Underestimated Pathogen in Veterinary Medicine in Italy. *German Journal Research*.

[63] Elfadadny A., Ragab R. F., AlHarbi M. (2024). Antimicrobial Resistance of *Pseudomonas aeruginosa*: Navigating Clinical Impacts, Current Resistance Trends, and Innovations in Breaking Therapies. *Frontiers in Microbiology*.

[64] Savin M., Sib E., Heinemann C. (2024). Tracing Clinically Relevant Antimicrobial Resistances in *Acinetobacter baumannii-calcoaceticus* Complex across Diverse Environments: A Study Spanning Clinical, Livestock, and Wastewater Treatment Settings. *Environment International*.

[65] Bortolaia V., Guardabassi L. (2023). Zoonotic Transmission of Antimicrobial-Resistant *Enterococci*: A Threat to Public Health or an Overemphasized Risk?. *Zoonoses: Infections Affecting Humans and Animals*.

[66] English E. L., Schutz K. C., Willsey G. G., Wargo M. J. (2018). Transcriptional Responses of *Pseudomonas aeruginosa* to Potable Water and Freshwater. *Applied and Environmental Microbiology*.

[67] Vukić Lušić D., Maestro N., Cenov A. (2021). Occurrence of *P. aeruginosa* in Water Intended for Human Consumption and in Swimming Pool Water. *Environments*.

[68] Grosso-Becerra M. V., Santos-Medellín C., González-Valdez A. (2014). *Pseudomonas aeruginosa* Clinical and Environmental Isolates Constitute a Single Population with High Phenotypic Diversity. *BMC Genomics*.

[69] Rehman S. (2023). A Parallel and Silent Emerging Pandemic: Antimicrobial Resistance (AMR) amid COVID-19 Pandemic. *Journal of Infection and Public Health*.

[70] Liffourrena A. S., Lucchesi G. I. (2014). Identification, Cloning and Biochemical Characterization of Pseudomonas Putida A (ATCC 12633) Monooxygenase Enzyme Necessary for the Metabolism of Tetradecyltrimethylammonium Bromide. *Applied Biochemistry and Biotechnology*.

[71] Lu Z., Mahony A. K., Arnold W. A., Marshall C. W., McNamara P. J. (2024). Quaternary Ammonia Compounds in Disinfectant Products: Evaluating the Potential for Promoting Antibiotic Resistance and Disrupting Wastewater Treatment Plant Performance. *Environmental Science: Advances*.

[72] Pereira A. P., Antunes P., Peixe L., Freitas A. R., Novais C. (2024). Current Insights into the Effects of Cationic Biocides Exposure on Enterococcus Spp. *Frontiers in Microbiology*.

[73] Zaheer R., Cook S. R., Barbieri R. (2020). Surveillance of *Enterococcus* Spp. Reveals Distinct Species and Antimicrobial Resistance Diversity across a One-Health Continuum. *Scientific Reports*.

[74] Pulami D., Schwabe L., Blom J. (2023). Genomic Plasticity and Adaptive Capacity of the Quaternary Alkyl-Ammonium Compound and Copper Tolerant Acinetobacter Bohemicus Strain QAC-21b Isolated from Pig Manure. *Antonie Van Leeuwenhoek*.

[75] Imran M., Das K. R., Naik M. M. (2019). Co-selection of Multi-Antibiotic Resistance in Bacterial Pathogens in Metal and Microplastic Contaminated Environments: An Emerging Health Threat. *Chemosphere*.

[76] Rajamohan G., Srinivasan V. B., Gebreyes W. A. (2010). Novel Role of A*cinetobacter Baumannii* RND Efflux Transporters in Mediating Decreased Susceptibility to Biocides. *Journal of Antimicrobial Chemotherapy*.

[77] Short F. L., Liu Q., Shah B. (2021). The *Acinetobacter baumannii* Disinfectant Resistance Protein, AmvA, Is a Spermidine and Spermine Efflux Pump. *Communications Biology*.

[78] Henderson P. J., Maher C., Elbourne L. D., Eijkelkamp B. A., Paulsen I. T., Hassan K. A. (2021). Physiological Functions of Bacterial “Multidrug” Efflux Pumps. *Chemical Reviews*.

[79] Hrovat K., Zupančič J. Č., Seme K., Avguštin J. A. (2023). QAC Resistance Genes in ESBL-Producing *E. coli* Isolated from Patients with Lower Respiratory Tract Infections in the Central Slovenia Region-A 21-Year Survey. *Tropical Medicine and Infectious Disease*.

[80] Sidhu M. S., Sørum H., Holck A. (2002). Resistance to Quaternary Ammonium Compounds in Food-Related Bacteria. *Microbial Drug Resistance*.

[81] Ibrahim W. A., Marouf S. A., Erfan A. M., Nasef S. A., El Jakee J. K. (2019). The Occurrence of Disinfectant and Antibiotic-Resistant Genes in *Escherichia coli* Isolated from Chickens in Egypt. *Veterinary World*.

[82] Enany M. E., Algammal A. M., Nasef S. A. (2019). The Occurrence of the Multidrug Resistance (MDR) and the Prevalence of Virulence Genes and QACs Resistance Genes in *E. coli* Isolated from Environmental and Avian Sources. *AMB Express*.

[83] Michaud M. E., Allen R. A., Morrison-Lewis K. R. (2022). Quaternary Phosphonium Compound Unveiled as a Potent Disinfectant against Highly Resistant *Acinetobacter baumannii* Clinical Isolates. *ACS Infectious Diseases*.

[84] Babaei M. R., Sulong A., Hamat R. A., Nordin S. A., Neela V. K. (2015). Extremely High Prevalence of Antiseptic Resistant Quaternary Ammonium Compound E Gene Among Clinical Isolates of Multiple Drug Resistant Acinetobacter Baumannii in Malaysia. *Annals of Clinical Microbiology and Antimicrobials*.

[85] Vijayakumar R., Sandle T., Al-Aboody M. S. (2018). Distribution of Biocide Resistant Genes and Biocides Susceptibility in Multidrug-Resistant *Klebsiella pneumoniae, Pseudomonas aeruginosa* and *Acinetobacter baumannii*—A First Report from the Kingdom of Saudi Arabia. *Journal of Infection and Public Health*.

[86] Fernandes L. M., Ramos G. L. D. P. A., Malta R. C. R., Nascimento J. d. S. (2022). Tolerance of Foodborne *Acinetobacter* Spp. To Sanitizer Agents. *The Journal of Infection in Developing Countries*.

[87] Chalbaud A., Ramos Y., Alonso G. (2012). Antibiotic and Disinfectant Resistance in *Acinetobacter baumannii* Genotyped Isolates from the Caracas University Hospital. *Microbes in Applied Research: Current Advances and Challenges*.

[88] Romão C. M. C. P. A., Faria Y. N. D., Pereira L. R., Asensi M. D. (2005). Susceptibility of Clinical Isolates of Multiresistant Pseudomonas aeruginosa to a Hospital Disinfectant and Molecular Typing. *Memórias do Instituto Oswaldo Cruz*.

[89] Subedi D., Vijay A. K., Willcox M. (2018). Study of Disinfectant Resistance Genes in Ocular Isolates of *Pseudomonas aeruginosa*. *Antibiotics*.

[90] Aase B., Sundheim G., Langsrud S., Rørvik L. M. (2000). Occurrence of and a Possible Mechanism for Resistance to a Quaternary Ammonium Compound in Listeria Monocytogenes. *International Journal of Food Microbiology*.

[91] Sundheim G., Langsrud S., Heir E., Holck A. L. (1998). Bacterial Resistance to Disinfectants Containing Quaternary Ammonium Compounds. *International Biodeterioration & Biodegradation*.

[92] Bland R., Waite-Cusic J., Weisberg A. J., Riutta E. R., Chang J. H., Kovacevic J. (2021). Adaptation to a Commercial Quaternary Ammonium Compound Sanitizer Leads to Cross-Resistance to Select Antibiotics in *Listeria Monocytogenes* Isolated from Fresh Produce Environments. *Frontiers in Microbiology*.

[93] Guo Z., Qin C., Zhang L. (2024). Distribution and Characterization of Quaternary Ammonium Biocides Resistant Bacteria in Different Soils, in South-Western China. *Microorganisms*.

[94] Chen S., Fu J., Zhao K. (2023). Class 1 Integron Carrying qacEΔ1 Gene Confers Resistance to Disinfectant and Antibiotics in *Salmonella*. *International Journal of Food Microbiology*.

[95] Nishihara T., Okamoto T., Nishiyama N. (2000). Biodegradation of Didecyldimethylammonium Chloride by Pseudomonas Fluorescens TN4 Isolated from Activated Sludge. *Journal of Applied Microbiology*.

[96] Han Y., Zhou Z. C., Zhu L. (2019). The Impact and Mechanism of Quaternary Ammonium Compounds on the Transmission of Antibiotic Resistance Genes. *Environmental Science and Pollution Research*.

[97] Nordholt N., Kanaris O., Schmidt S. B., Schreiber F. (2021). Persistence against Benzalkonium Chloride Promotes Rapid Evolution of Tolerance during Periodic Disinfection. *Nature Communications*.

[98] Hassan K. A., Baltzer S., Paulsen I., Brown M. (2010). Pumping Out Biocides–Cause for Concern. *Microbiology Australia*.

[99] Tezel U. (2009). *Fate and Effect of Quaternary Ammonium Compounds in Biological Systems*.

[100] White D. G., McDermott P. F. (2001). Emergence and Transfer of Antibacterial Resistance. *Journal of Dairy Science*.

[101] Kumar A., Schweizer H. P. (2005). Bacterial Resistance to Antibiotics: Active Efflux and Reduced Uptake. *Advanced Drug Delivery Reviews*.

[102] Poole K. (2007). Efflux Pumps as Antimicrobial Resistance Mechanisms. *Annals of Medicine*.

[103] Slipski C. J., Zhanel G. G., Bay D. C. (2018). Biocide Selective TolC-independent Efflux Pumps in Enterobacteriaceae. *Journal of Membrane Biology*.

[104] Piddock L. J. (2006). Multidrug-resistance Efflux Pumps? Not Just for Resistance. *Nature Reviews Microbiology*.

[105] Grkovic S., Brown M. H., Skurray R. A. (2002). Regulation of Bacterial Drug Export Systems. *Microbiology and Molecular Biology Reviews*.

[106] Buffet-Bataillon S., Tattevin P., Bonnaure-Mallet M., Jolivet-Gougeon A. (2012). Emergence of Resistance to Antibacterial Agents: the Role of Quaternary Ammonium Compounds—A Critical Review. *International Journal of Antimicrobial Agents*.

[107] Bragg R. R., Meyburgh C. M., Lee J. Y., Coetzee M. Potential Treatment Options in a Post-antibiotic Era.

[108] Mc Cay P. H., Ocampo-Sosa A. A., Fleming G. T. (2010). Effect of Subinhibitory Concentrations of Benzalkonium Chloride on the Competitiveness of Pseudomonas aeruginosa Grown in Continuous Culture. *Microbiology*.

[109] Tezel U., Pavlostathis S. G. (2011). Role of Quaternary Ammonium Compounds Resistance in on the Antimicrobial Environment. *Antimicrobial Resistance in the Environment*.

[110] Gaze W. H., Zhang L., Abdouslam N. A. (2011). Impacts of Anthropogenic Activity on the Ecology of Class 1 Integrons and Integron-Associated Genes in the Environment. *The ISME Journal*.

[111] Alav I., Sutton J. M., Rahman K. M. (2018). Role of Bacterial Efflux Pumps in Biofilm Formation. *Journal of Antimicrobial Chemotherapy*.

[112] Hajiagha M. N., Kafil H. S. (2023). Efflux Pumps and Microbial Biofilm Formation. *Infection, Genetics and Evolution*.

[113] Levy S. B. (2002). Factors Impacting on the Problem of Antibiotic Resistance. *Journal of Antimicrobial Chemotherapy*.

[114] Liu C., Goh S. G., You L. (2023). Low Concentration Quaternary Ammonium Compounds Promoted Antibiotic Resistance Gene Transfer via Plasmid Conjugation. *The Science of the Total Environment*.

[115] Stewart P. S. (2002). Mechanisms of Antibiotic Resistance in Bacterial Biofilms. *International journal of medical microbiology*.

[116] Bridier A., Briandet R., Thomas V., Dubois-Brissonnet F. (2011). Resistance of Bacterial Biofilms to Disinfectants: a Review. *Biofouling*.

[117] Maurice N. M., Bedi B., Sadikot R. T. (2018). *Pseudomonas aeruginosa* Biofilms: Host Response and Clinical Implications in Lung Infections. *American Journal of Respiratory Cell and Molecular Biology*.

[118] Pang Z., Raudonis R., Glick B. R., Lin T. J., Cheng Z. (2019). Antibiotic Resistance in *Pseudomonas aeruginosa*: Mechanisms and Alternative Therapeutic Strategies. *Biotechnology Advances*.

[119] Qin S., Xiao W., Zhou C. (2022). *Pseudomonas aeruginosa*: Pathogenesis, Virulence Factors, Antibiotic Resistance, Interaction with Host, Technology Advances and Emerging Therapeutics. *Signal Transduction and Targeted Therapy*.

[120] Lineback C. B., Nkemngong C. A., Wu S. T., Li X., Teska P. J., Oliver H. F. (2018). Hydrogen Peroxide and Sodium Hypochlorite Disinfectants Are More Effective against *Staphylococcus aureus* and *Pseudomonas aeruginosa* Biofilms Than Quaternary Ammonium Compounds. *Antimicrobial Resistance and Infection Control*.

[121] Abdallah M., Khelissa O., Ibrahim A. (2015). Impact of Growth Temperature and Surface Type on the Resistance of *Pseudomonas aeruginosa* and *Staphylococcus aureus* Biofilms to Disinfectants. *International Journal of Food Microbiology*.

[122] Giaouris E., Chorianopoulos N., Doulgeraki A., Nychas G. J. (2013). Co-culture with *Listeria Monocytogenes* within a Dual-Species Biofilm Community Strongly Increases Resistance of *Pseudomonas Putida* to Benzalkonium Chloride. *PLoS One*.

[123] Pang X. Y., Yang Y. S., Yuk H. G. (2017). Biofilm Formation and Disinfectant Resistance of Salmonella Sp. In Mono‐and Dual‐species with *Pseudomonas aeruginosa*. *Journal of Applied Microbiology*.

[124] Cycoń M., Mrozik A., Piotrowska-Seget Z. (2019). Antibiotics in the Soil Environment—Degradation and Their Impact on Microbial Activity and Diversity. *Frontiers in Microbiology*.

[125] Jiao Y., Niu L. N., Ma S., Li J., Tay F. R., Chen J. H. (2017). Quaternary Ammonium-Based Biomedical Materials: State-Of-The-Art, Toxicological Aspects and Antimicrobial Resistance. *Progress in Polymer Science*.

[126] Garcıa M. t, Ribosa I., Guindulain T., Sanchez-Leal J., Vives-Rego J. (2001). Fate and Effect of Monoalkyl Quaternary Ammonium Surfactants in the Aquatic Environment. *Environmental Pollution*.

[127] Jing G., Zhou Z., Zhuo J. (2012). Quantitative Structure-Activity Relationship (QSAR) Study of Toxicity of Quaternary Ammonium Compounds on Chlorella Pyrenoidosa and Scenedesmus Quadricauda. *Chemosphere*.

[128] van der Meer J. (2006). Metabolic Theories in Ecology. *Trends in Ecology & Evolution*.

[129] Belter B., McCarlie S. J., Boucher-van Jaarsveld C. E., Bragg R. R. (2022). Investigation into the Metabolism of Quaternary Ammonium Compound Disinfectants by Bacteria. *Microbial Drug Resistance*.

[130] Jiang J., Ding X., Tasoglou A. (2021). Real-Time Measurements of Botanical Disinfectant Emissions, Transformations, and Multiphase Inhalation Exposures in Buildings. *Environmental Science and Technology Letters*.

[131] Tandukar M., Oh S., Tezel U., Konstantinidis K. T., Pavlostathis S. G. (2013). Long-Term Exposure to Benzalkonium Chloride Disinfectants Results in Change of Microbial Community Structure and Increased Antimicrobial Resistance. *Environmental Science and Technology*.

[132] Brayton S. R., Toles Z. E., Sanchez C. A. (2023). Soft QPCs: Biscationic Quaternary Phosphonium Compounds as Soft Antimicrobial Agents. *ACS Infectious Diseases*.

[133] Sommers K. J., Michaud M. E., Hogue C. E. (2022). Quaternary Phosphonium Compounds: an Examination of Non-nitrogenous Cationic Amphiphiles that Evade Disinfectant Resistance. *ACS Infectious Diseases*.

